# Polarization Enhanced Charge Transfer: Dual-Band GaN-Based Plasmonic Photodetector

**DOI:** 10.1038/srep40483

**Published:** 2017-01-13

**Authors:** Ran Jia, Dongfang Zhao, Naikun Gao, Duo Liu

**Affiliations:** 1 State Key Laboratory of Crystal Materials, Shandong University, 27 South Shanda Road, Jinan, Shandong 250100, P. R. China

## Abstract

Here, we report a dual-band plasmonic photodetector based on Ga-polar gallium nitride (GaN) for highly sensitive detection of UV and green light. We discover that decoration of Au nanoparticles (NPs) drastically increases the photoelectric responsivities by more than 50 times in comparition to the blank GaN photodetector. The observed behaviors are attributed to polarization enhanced charge transfer of optically excited hot electrons from Au NPs to GaN driven by the strong spontaneous polarization field of Ga-polar GaN. Moreover, defect ionization promoted by localized surface plasmon resonances (LSPRs) is also discussed. This novel type of photodetector may shed light on the design and fabrication of photoelectric devices based on polar semiconductors and microstructural defects.

Gallium nitride (GaN) has extensive applications in next generation electronics such as field effect transistors (FET)^1^, light emitting diodes (LED)^2^, lasers^3^, solar cells^4^ photodetectors^5^ and other high-power devices^6^,^7^. GaN-based photodetectors are considered to be ideal candidates for detection of UV light (wavelength λ < 365 nm). As a typical polar semiconductor, GaN consists of sequential hexagonal bilayers made of Ga^3+^ and N^3−^ ions, respectively. The lack of inversion symmetry in GaN gives rise to a large spontaneous polarization charge of ~ 0.029 C/m^2 ^8 and an electric field of ~10^7^ V/cm^9^, pointing from the Ga-terminated surface to the N-terminated surface. The polarization filed is very strong and can greatly influence the performance of GaN-based electronic and optoelectronic devices by band tilt and charge accumulation at the heterostructure interfaces, e.g. AlGaN/GaN^10^, InGaN/GaN^11^ and Shottkey diodes^12^.

Plasmonic nanostrucutres, which support localized surface plasmon resonances (LSPRs), collective oscillation of conduction band electrons, have been widely used to tune the optoelectronic process in photovoltaics^13^,^14^, photocatalysis^15^, and photodetectors^16^ through enhanced optical absorption and/or charge transfer^17^,^18^. The electromagnetic decays of LSPRs usually take place through either radiation of photons or through non-radiative transfer of energy to hot electrons. The non-radiative decay takes place through intraband or interband excitations of hot electrons^19^. Note that, for Au nanoparticles (NPs), hot electrons could also be obtained through interband transition of electrons by UV excitation. The hot electrons could then be collected by a nearby active component, e.g. a semiconductor, or converted into heat through electron-electron and lattice collisions. However, the emission of hot electrons into a nearby semiconductor was believed to have a low efficiency (~0.01%)^20^,^21^. Is it possible to use the polarization field of a polar semiconductor to improve the collection efficiency of hot electrons? In this article, we explored the possibility of high yield injection of hot electrons into GaN, a model polar semiconductor, for the development of high performance photodetector.

## Method

### Sample preparation

The Ga-polar GaN (0001) wafers used in this study were grown on sapphire substrates using the hydride vapor phase epitaxy (HVPE) method (Nanowin, China). The GaN films are ~4 μm thick, n-type in nature, and unintentionally doped with a carrier concentration of ~10^17^/cm^3^. To fabricate the photodetector, the wafers were first supersonically washed sequentially with acetone, ethanol and deionized water in sequence for 15 min each. They were then coated with a thin layer of gold in vacuum using a sputtering coater (ETD 2000, China) for 20 s at 10 mA, followed by annealing at elevated temperatures. After Au NPs were prepared, coplanar interdigitated electrodes (finger width 200 μm and spacing 200 μm) were fabricated by using a metal mask to measure the photoelectric responsivities. A blank sample and two samples annealed at 300 °C and 500 °C for 1 h were selected to illustrate the results (abbreviated as Blk, Au300C and Au500C, respectively).

### Characterization

**Figure 1:**
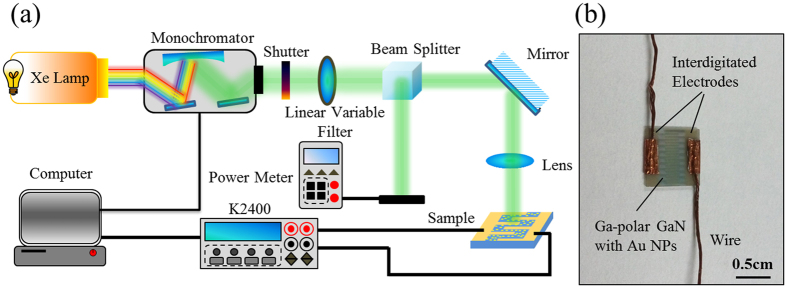
Schematic of experimental setup used for the measurement of photoelectric responsivity **(a)** and a photograph of the photodetectors **(b)**.

Figure 1 shows the experimental setup used for the measurement of photoelectric responsivity and a photograph of the photodetectors. The light source consists of a 150 W Xe lamp and a monochromator (Omni-λ150, Zolix, China). The light beam was split by a beam splitter into two beams of equal intensity. One was tuned and reflected onto the samples as the excitation source, and the other served as the reference and was monitored by an optical power meter (PD300-70 UV-193, Ophir Optronics, USA). The samples were stored in dark for at least 24 h prior to testing. The photoelectric response curves were recorded by a source meter (Keithley 2400, USA) under a bias voltage of 0.8 V with illumination from 355 nm to 615 nm (step = 10 nm) at a constant optical power of 30 μW. The current-voltage (I-V) curves were obtained by sweeping the voltage from −1.0 V to 1.0 V under UV (365 nm) and green light (525 nm) illuminations, respectively. The photocurrent-power curves were obtained by tuning the optical power from 10 μW to 100 μW. The photocurrent-time curves were recorded with light on and off every 10 s.

The morphologies of the three samples (Blk, Au300C and Au500C) were examined using scanning electron microscopy (SEM, Hitachi S-4800, Japan) and atomic force microscopy (AFM, Veeco Dimension Icon, USA). The surface chemical environments of Au500C were studied by X-ray photoelectron spectrometer (XPS, Thermo Scientific ESCALAB 250, Germany) equipped with a monochromatic Al Kα excitation source. The reflection spectra were measured using a spectrophotometer (UV1901PC, China). The photoluminescence (PL) spectra were obtained at room temperature by a spectrofluorometer (PI-PLE-2355/2558+PIXIS:256E, USA) equipped with a 325 nm He-Cd laser as the excitation source.

## Results and Discussion

### Morphology

**Figure 2:**
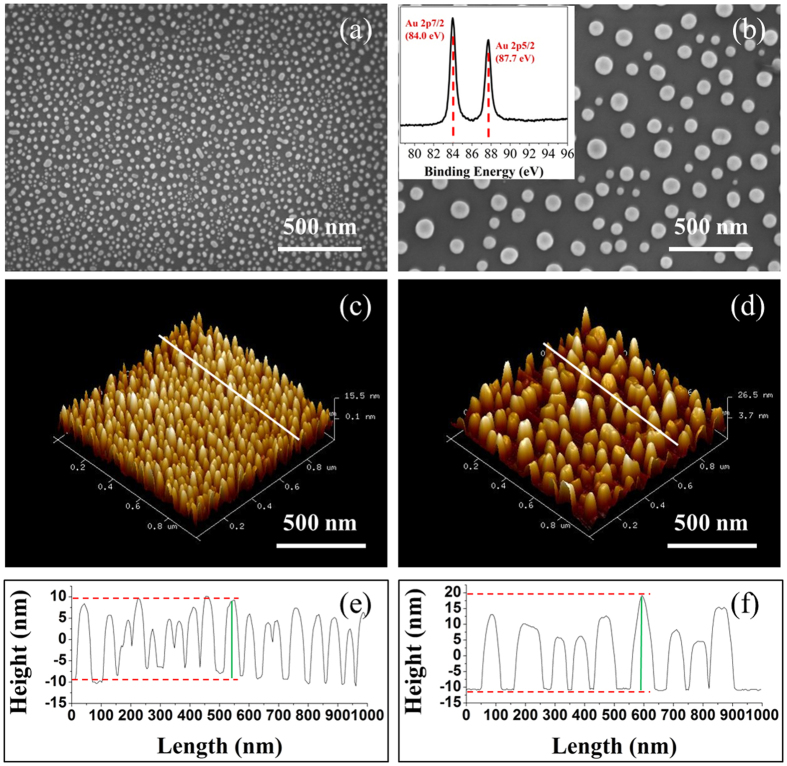
The SEM and AFM images and typical line profiles of Au300C **(a, c, e)** and Au500C **(b, d, f)**. Inset in (**b**) is the Au 4 f XPS spectrum for Au500C.

Figure 2 shows the SEM and AFM images and typical line profiles for Au300C and Au500C. Both samples were covered by irregular hemispherical Au NPs. The Au 4 f XPS spectrum for Au 500 C indicates that the Au NPs remain unoxidized, similar to previous investigations^22^. Higher annealing temperature reduces the coverage ratio and increases Au NPs sizes. The growth kinetics of Au NPs agrees with previous investigation for metal nanoparticles on flat surface, which usually follow two principles through coalescence and/or Ostwald ripening^23^,^24^. The microstructural characteristics for both samples are summarized in Table 1. The average heights for Au NPs were obtained from the AFM profiles, while the average diameters, densities, and the coverage ratios for Au NPs were obtained by image analysis of the SEM images.

### Optical and Electrical Properties

**Figure 3:**
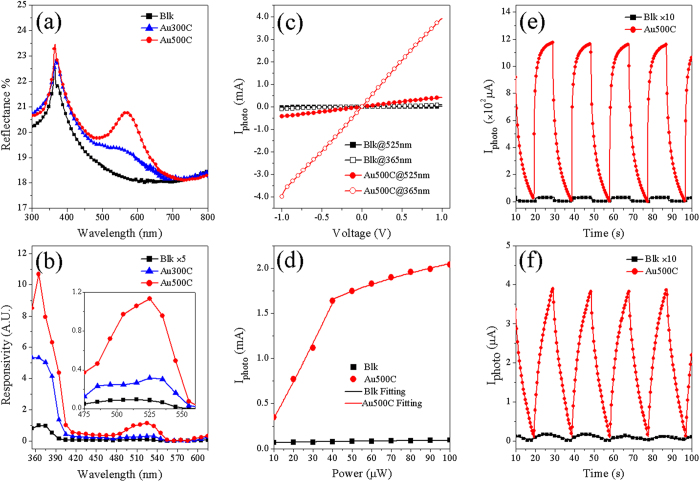
Optical and electrical properties of the three samples (Blk, Au300C and Au500C). **(a)** reflectance spectra; **(b)** photoelectric response curves (inset: enlarged view around the green light region) recorded under a bias voltage of 0.8 V at a constant optical power of 30 μW; **(c)** I-V curves of Blk and Au500C under UV (365 nm) and green light (525 nm) illuminations, respectively; **(d)** photocurrents for Blk and Au500C under green light illumination as a function of incident optical power; **(e, f)** photocurrent-time curves with light on and off every 10 s for UV and green light, respectively.

We investigated the optical and photoelectric properties of the three samples. Figure 3a shows the optical reflectance spectra obtained after calibration with a standard Al foil. The reflectance peaks at 365 nm correspond to the band edge of GaN (*E*_*g*_ = 3.4 eV). The presence of Au NPs increases reflectance in the visible region due to enhanced backward scattering by Au NPs. The broad peaks in green light region can be attributed to the dipole mode of LSPRs of Au NPs^25^. The LSPR peak for Au500C is more pronounced due to higher scattering efficiency of larger Au NPs^26^.

Figure 3b shows the photoelectric responsivity (PR, defined as photocurrent divided by incident optical power) of the samples from 355 nm to 615 nm. Au300C and Au500C exhibit two responsive peaks in the UV and green light region with greatly enhanced photoelectric responsivities than the blank sample. The enhancement ratios for UV light are ~27 and ~54 times, while enhancement ratios for green light are ~18 and ~64 times. As the Au NPs were obtained by annealing at elevated temperatures, possible effects of thermal treatment should be considered. Thus, we measured the photoelectric response of two blank photodetectors obtained by annealing at 500 °C for 1 h in vacuum. The measured dark currents and photocurrents reveal very little difference from the sample without annealing, which suggests that the defect structures of GaN (grown at 1100 °C) will not be changed by annealing at 500 °C.

Figure 3c shows the I-V curves of the blank sample and Au500C under UV and green light illumination, which shows an Ohmic like behavior. The slopes of Au500C in the UV and green light region are ~54 and ~64 times greater, respectively, than those of the blank sample, in agreement with the photoelectric response results, further demonstrating the impact of Au NPs on increased the photocurrents. Figure 3d shows the photocurrent-power curves for the blank sample and Au500C under green light illumination with the optical power varying from 10 μW to 100 μW. The photocurrents for the blank sample increase slightly with the optical power, while the photocurrents for Au500C increase drastically and start to saturate after the power reaches 40 μW. Figure 3e and 3f show the photocurrent-time curves obtained by turning the light sources on and off every 10 s. The photocurrents for Au500C are much greater than that of the blank sample. Notably, the rise/fall times are smaller for UV (2.9 s/6.2 s) than that for green light (7.7 s/8.2 s). The experimental data can be fitted by 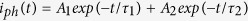
27, where *τ*_1_ and *τ*_2_ are the time constants and A_1_ and A_2_ are the fitting constants for the slow and fast components, respectively. It should be noted that reducing the interdigitated finger spacing will also reduce the response time.

### Mechanism for the Dual-Band Detection

**Figure 4:**
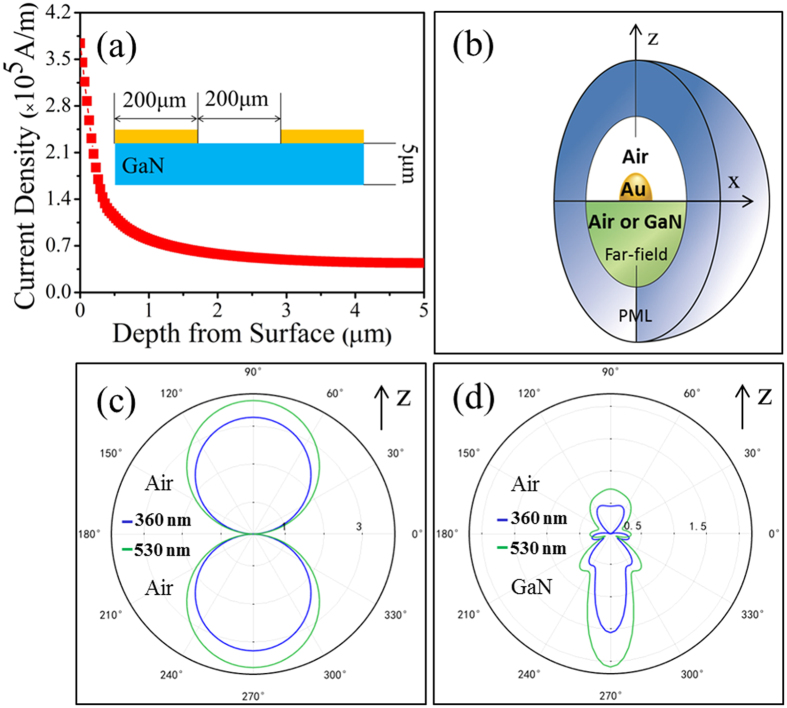
(**a**) Current density distribution as a function of depth for a coplanar GaN photodetector with the geometry shown in the inset. **(b)** Schematic illustration of the FEM model used for simulation around a hemispherical Au NP. **(c)** Scattering efficiency of a hemispherical Au NP (*r* = 25 nm) in air under illumination at 360 nm and 530 nm, respectively. **(d)** Scattering efficiency for the Au NP (*r* = 25 nm) on GaN under illumination at 360 nm and 530 nm, respectively.

For a coplanar photodetector, most of the photocurrent is generated from the near surface region. We perform finite element method simulation (FEM, Comsol Multiphysics) to examine the current distribution for a blank coplanar GaN photodetector with the same geometry and a bias voltage of 0.8 V, identical to the parameters used in our experiments. The resistivity of GaN was set to be 0.05 Ω∙cm. As expected, the current density decreases exponentially from the surface to the bottom, as shown in Fig. 4a. An integration of the data reveals that the current in the top 100 nm layer of the GaN is ~7.5 times greater than that in the bottom.

Since Au NPs can concentrate light into the near surface region of a dielectric^28^,^29^, we perform FEM calculations of the scattering efficiency of a hemispherical Au NP (*r* = 25 nm). We chose 360 nm and 530 nm as the typical wavelengths for UV and green light, respectively. Figure 4b shows the spherical computation domain surround by a perfectly matched layer (PML). The thickness of the PML is set to be half of the incident wavelength. The maximum mesh size (MMS) in the Au NP is set to 5 nm, while the MMS in other domains is set to 1/10 of the incident wavelength. The light is represented by a plane wave beam transmitted in the negative *z* direction and polarized along the *x* axis. The background field was calculated according to the Fresnel law. Due to the symmetric boundary conditions, only half of the domain was calculated. The scattering efficiency for the Au NP in air resembles that of a dipole, characterized by identical forward and backward scattering profiles (Fig. 4c). In contrast, the scattering efficiency for the Au NP on GaN is quite different. The Au NP scatters most incident light forward into GaN (Fig. 4d) due to high density optical modes in GaN because light scattered with high in-plane wave-vectors that are evanescent in air can propagate in GaN^30^. Note that the scattering efficiency for 530 nm is greater than that for 360 nm.

**Figure 5:**
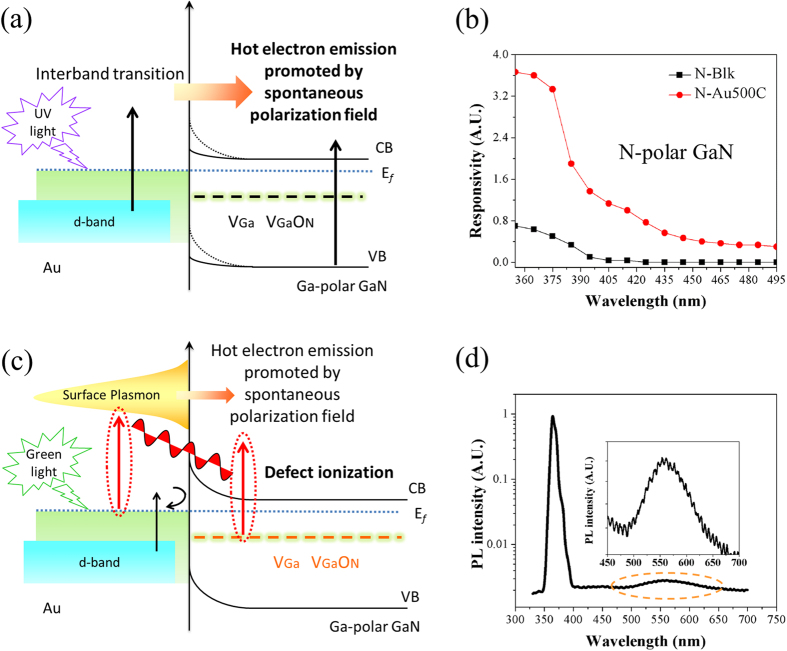
(**a**) Schematic illustration of the interband transition with hot electrons injection for enhanced photoelectric responsivity to UV light. **(b)** Photoelectric response curves in UV region for two N-polar samples (N-Blk and N-Au500C). **(c)** Schematic illustration of coupling between LSPRs of Au NPs and defect for enhanced photoelectric responsivity to green light. **(d)** The room temperature PL spectrum of a blank GaN wafer under excitation by a 325 nm He-Cd laser (inset: an enlarged view in the visible region).

We then estimate the photocurrent enhancement ratio by integrating the energy density (Fig. 4d) over the near surface region multiplied by the current density curve (Fig. 4a) under the assumption of a 100% photon-to-electron conversion efficiency for both samples with and without Au NPs. Our calculation reveals that the Au NPs can increase the photoelectric responsivity by 4.4 and 3.1 times for 360 nm and 530 nm, respectively, both of which are smaller than the measured values. A literature review reveals that plasmonic photodetectors fabricated on non-polar semiconductors usually exhibit low photoelectric responsivity, in particular reduced responsivities in the UV region^31^,^32^,^33^. Consequently, we propose that the enhanced photoelectric response could originate from high yield injection of optically excited hot electrons^34^ by the polarization field of GaN. The hot electrons come from interband transition of the filled *d*-band electrons of Au NPs (with its upper edge ~ 2.3 eV below the Fermi level) upon UV illumination, as shown in Fig. 5a.

This new principle based on polarization enhanced charge transfer was verified experimentally on N-polar GaN due to the difference in the direction and magnitude of the electric field from Ga-polar GaN^12^. The N-polar GaN used in our study were free standing wafers with similar doping concentrations and conductance to the Ga-polar GaN. A blank sample and a sample covered with Au NPs obtained under the same experimental procedures as Au500C were selected to illustrate the results (abbreviated as N-Blk and N-Au500C, respectively). Figure 5b shows the photoelectric response curves for both samples. The photoelectric responsivity of N-Au500C to UV light is ~4 times of the blank sample, much smaller than the enhancement ratio for the Ga-polar sample. We also estimate the built-in electric field, *E*_*bi*_, for the Au/GaN interface by using the classical model for Schottky barrier^35^ given by *E*_*bi*_ = *Φ*_*B*_*/W*, where *Φ*_*B*_ is the Schottky barrier height and *W* is the width of the depletion layer. *E*_*bi*_ was calculated to be <10^5^ V/cm, much smaller than the spontaneous polarization field of GaN (~10^7^ V/cm). Therefore, we believe that the polarization field does play a significant role in the collection of hot electrons, similar to hot carrier collection in metal–insulator–semiconductor (MIS) heterostructures^36^.

The enhanced photoelectric responsivity for green light is different from that for UV light. Besides the polarization enhanced charge transfer, the saturation of photocurrent (Fig. 3d) suggests the charge carriers originate from the ionized defects, as shown in Fig. 5c. Microstructural defects, such as point defects, impurities, and their complexes, are usually abundant in GaN wafers^37^,^38^,^39^. Their presence breaks the local crystallographic symmetry, creates additional energy levels within the band gap, and enables optical absorption and emission in the visible region. Figure 5d shows a typical PL spectrum of the blank GaN sample characterized by a broad green band. The green band is closely linked to the gallium vacancy (*V*_*Ga*_) and its complex with substitutional oxygen (*V*_*Ga*_ − *O*_*N*_)^40^,^41^, which are the dominant defects in n-type GaN^42^. The 30 nm difference can be attributed to defect ionization, as discussed above. Note that only forward scattered light with energy greater than the defect levels can promote defect ionization which contributes to the photoelectric response.

We use the classical theory for a coplanar photoconductor^43^ to calculate the carrier generation rate (*G*) of the plasmonic photodetector. For an optical power of 40 μW at 525 nm, the number of photons that reach the photodetector per second is 1.06 × 10^14^, corresponding to ~6.6 × 10^9^/cm^2^ for a carrier lifetime of ~10 μs^42^,^44^ greater than the defect density (~1.6 × 10^9^/cm^2^) for our samples. Therefore, we expect that all *V*_*Ga*_ and *V*_*Ga*_ − *O*_*N*_ in the top surface region of the photodetector have been ionized, resulting in an external quantum efficiency (EQE) of ~27.6% for Au500C, possibly resulted from coupling between LSPRs and the defect levels in a way similar to the model recently proposed by Lian *et al*. for CdSe-Au nanorods (NRs) with a very high transient quantum yield (>24%)^45^ or that proposed by Hwang *et al*. for ZnO-Au NRs^46^.

## Conclusion

We have developed a dual band plasmonic photodetector on GaN for detection of UV and green light, respectively. We discover that Au NPs covered on Ga-polar GaN can drastically enhance the photoelectric responsivities by ~50 and ~60 times in response to UV and green light, respectively. The enhancement ratios are much greater than the values obtained by FEM simulation, 4.4 and 3.1 times for UV and green lights, respectively, on the photodetectors with the same geometry. A comparative study of similar photodetectors on N-polar GaN reveals a reduced enhancement ratio of ~4. It is concluded that polarity plays an essential role on the measured photocurrent and Ga-polar surface can greatly increase collection efficiency of hot electrons in plasmonic nanostructures upon optical excitation. Possible coupling between LSPR and microstructural defects are also discussed. This novel design principle offers a new avenue for adopting polar semiconductors to high performance optoelectronic devices.

## Additional Information

**How to cite this article**: Jia, R. *et al*. Polarization Enhanced Charge Transfer: Dual-Band GaN-Based Plasmonic Photodetector. *Sci. Rep.* **7**, 40483; doi: 10.1038/srep40483 (2017).

**Publisher's note:** Springer Nature remains neutral with regard to jurisdictional claims in published maps and institutional affiliations.

## Figures and Tables

**Table 1:** Microstructural characteristics of Au NPs on GaN after annealing at 300 °C and 500 °C.

Sample	Average height (nm)	Average diameter (nm)	Density (/μm^2^)	Coverage ratio (%)
Au300C	15.1 ± 2.2	22.2 ± 0.4	653 ± 19	25.4 ± 1.6
Au500C	20.6 ± 4.9	67.3 ± 4.9	56 ± 9	20.0 ± 2.7
